# The Potential Coexistence of Autoimmune Thyroid Diseases and Pediatric Vulvar *Lichen sclerosus*

**DOI:** 10.3390/children11020255

**Published:** 2024-02-16

**Authors:** Agnieszka Dulska, Jakub Bodziony, Marta Janik, Agnieszka Drosdzol-Cop

**Affiliations:** 1 Chair and Department of Gynecology, Obstetrics and Oncological Gynecology, Faculty of Medical Sciences in Katowice, Medical University of Silesia in Katowice, Markiefki 87, 40-211 Katowice, Poland; adrosdzol@sum.edu.pl; 2 Department of Physiology, Faculty of Medical Sciences in Katowice, Medyków 18, 40-752 Katowice, Poland; 3 Euroimmun Polska Sp. z o.o., 50-543 Wrocław, Poland; m.janik@euroimmun.pl

**Keywords:** adolescent, autoimmune thyroid disease, vulvar *Lichen sclerosus*

## Abstract

Introduction: Vulvar lichen sclerosus (VLS), a chronic inflammatory skin disorder, often coexists with autoimmune thyroid disease (AITD). VLS presents with subtle symptoms including vulvar itching and discomfort. Clinically, a “Figure 8” pattern involving the labia minora, clitoral hood, and perianal region is often observed. It is prevalent both in pre-pubertal girls and women aged 40–60, and the link between VLS and AITD remains unclear, with proposed causes including autoimmune, hormonal or genetic factors, and environmental triggers. This study addresses the lack of research on the association in children, aiming to investigate the largest group of underage girls to date. Aim: This study aimed to investigate the coexistence of thyroid autoimmune diseases in girls diagnosed with vulvar lichen sclerosus (VLS) and to assess the presence of antibodies for specific thyroid autoimmune diseases. Materials and Methods: Our study was conducted from July 2020 to February 2021, involving a sample of 55 girls aged 2–18 years old, all free from systemic diseases. The study group comprised 20 girls previously diagnosed with vulvar lichen sclerosus (VLS), while the control group included 35 girls without VLS. Legal guardians completed questionnaires detailing the medical history of their children. Blood samples were collected from all participants and subjected to biochemical analysis. The presence of human IgG antibodies against thyroid peroxidase and IgG antibodies against thyroglobulin was assessed using the immunoenzymatic method with commercially available ELISA kits. Results: In the study group, common symptoms included itching, soreness, burning sensation, excoriation, and erythema or pallor of the skin and perineal mucosa. An evaluation of anti-thyroglobulin and anti-thyroid peroxidase antibodies revealed no statistical significance between the study and control groups (anti-TG *p* = 0.379, anti-TPO *p* = 0.96). Family history of autoimmune diseases showed no significant correlation with anti-thyroid antibodies in girls. Although no significant relation between VLS occurrence and antibody levels was found, it emphasizes the need for multidisciplinary medical care. Further research with larger patient groups is necessary.

## 1. Introduction

Vulvar lichen sclerosus (VLS) is a chronic inflammatory skin disorder, often associated with an increased risk for autoimmune thyroid disease (AITD). VLS affects approximately 1:900 of premenarchal girls [1,2] and is a chronic condition; however, the course is usually benign and self-limiting. The majority of affected patients present with subtle symptoms that are often misinterpreted as mild dermatitis. The inflammation associated with the condition results in the thickening of the skin on the vulva and surrounding structures. The most frequently observed symptoms associated with the disease include vulvar itching, discomfort, painful urination (dysuria), and constipation. During physical examinations, clinicians often identify a characteristic “Figure 8” pattern, which involves the labia minora, clitoral hood, and the perianal area. The symptoms of VLS, even though mostly mild, significantly reduce the quality of life [2].

VLS can manifest at any age or in any gender. Nevertheless, the highest prevalence is typically found in women between the ages of 40 and 60, as well as in pre-pubertal girls. Notably, there is a distinct peak in incidence among girls aged 4 to 6, representing 7–15% of all reported cases of vulvar lichen sclerosus [3].

Several clinical, histological, and serological studies have found that the prevalence of AITD is increased in patients with VLS; therefore, there is a growing body of evidence that suggests a link between the two conditions. AITD typically presents with a variety of nonspecific symptoms that include fatigue, joint pain, weight loss, heat intolerance, and shortness of breath [4,5,6,7,8].

The most common autoimmune thyroid diseases are Graves’ disease (GD), affecting approximately 3% of women, and Hashimoto’s disease, affecting 5%. Diagnosing Hashimoto’s disease and Graves’ disease typically involves a combination of medical history, physical examination, and laboratory tests. Thyroid hormone levels (TSH, T3, and T4) are measured, along with anti-thyroid peroxidase (anti-TPO) or anti-thyroglobulin antibodies (anti-TG). Elevated TSH and low T3 and T4 levels are common in Hashimoto’s disease. In contrast, for Graves’ disease, TSH levels are usually low, while T3 and T4 levels are high. Additionally, thyroid-stimulating immunoglobulins (TSIs), thyrotropin receptor antibodies (TRAbs), and anti-thyroid peroxidase (anti-TPO) are evaluated, and these antibodies are often elevated in Graves’ disease [9,10].

Currently, there is no proven correlation between VLS and AITD; however, recent studies show that 15–34% of cases in adult women and 14% in girls coexist with allergies or autoimmune diseases, such as the following: vitiligo, thyroiditis, type 1 diabetes mellitus, alopecia areata, or celiac disease [1]. The cause of the association between VLS and AITD is unknown, but several theories have been proposed to explain this association. These theories include environmental triggers, genetic factors, and immune dysregulation. 

So far, no one has examined the connection between AITD and children diagnosed with vulvar lichen sclerosus, although coexistence is suggested. According to our best knowledge, this is the largest study on a population of underage girls so far.

## 2. Aim

The aim of this research project is to investigate the coexistence of autoimmune thyroid diseases in girls diagnosed with vulvar lichen sclerosus (LS) and to assess the presence of antibodies characteristic of specific autoimmune diseases of the thyroid gland, in order to provide the patient with multidisciplinary medical care. 

## 3. Materials and Methods

### 3.1. Patients 

Our study sample was derived from the gynecological clinic Centrum Zdrowia Kobiety in Katowice, Poland, between July 2020 and February 2021. Twenty girls diagnosed with vulvar lichen sclerosus were recruited for the study (n = 20), and the control group consisted of 35 girls without diagnosed vulvar lichen sclerosus, coming to the clinic due to conditions such as genital infections or labial synechiae. 

The inclusion criteria were as follows: aged 2-18 years old; diagnosed lichen sclerosus of the vulva (diagnosis in children based on medical history and physical examination); no systemic diseases (e.g., cardiovascular diseases, peptic ulcer disease, epilepsy); and consent of the girl and/or her legal guardian to participate in the study. 

The exclusion criteria comprised the following: previously diagnosed autoimmune disease; pharmacotherapy used in the last 6 months (including hormonal drugs, contraceptives, NSAIDs); systemic diseases (e.g., cardiovascular diseases, peptic ulcer disease, epilepsy, liver and kidney diseases); addiction; pregnancy (current or in history); and a lack of consent from the girl and/or her legal guardian to participate in the study.

The project received a positive opinion from the Ethics Committee of the Medical University of Silesia in Katowice, Poland, obtaining the experiment approval no. KNW/0022/KB1/5/19. The project was implemented as a statutory work of the Medical University of Silesia, No. KNW-1-142/N/8/K.

### 3.2. Anamnesis 

For each patient, a questionnaire was completed on the basic demographic data of the patient, the course of the neonatal period, lifestyle, a gynecological interview, and family history, with particular emphasis on the presence of autoimmune diseases in the family. Patients from the study group also answered questions about their underlying disease—first symptoms, diagnosis, and treatment and its effect. 

### 3.3. Blood Collection

Blood samples (10 mL) were collected from the ulnar vein, in the morning, before breakfast. Blood was collected in standard blood tubes with EDTA (1.6 mg/mL EDTA- K3; S-Monovette, SARSTEDT). The samples for serum analysis were centrifuged at 4000× *g* rpm for 10 min at 4 °C and stored at −80 °C. Plasma and serum samples were subsequently frozen and stored at −80 °C until biochemical analyses could be performed.

### 3.4. Anti-TG Assessment

Human IgG antibodies against thyroglobulin (anti-TG) plasma concentrations were assessed in duplicates by the immunoenzymatic method with the commercially available ELISA kits (Euroimmun, Poland) (kit no. EA 1013-9601 G). Qualitative and quantitative assessments were carried out. Values below 100 IU/mL were negative results, while values equal to and higher than 100 IU/mL were positive results.

### 3.5. Anti-TPO Assessment

Human IgG A antibodies against thyroid peroxidase (anti-TPO) plasma concentrations were assessed in duplicates by the immunoenzymatic method with the commercially available ELISA kits (Euroimmun, Poland) (kit no. EA 1012-9601 G). Qualitative and quantitative assessments were carried out. Values below 50 IU/mL were negative results, while values equal to and higher than 50 IU/mL were positive results.

### 3.6. Statistical Analysis

A statistical analysis was performed using STATISTICA 12.5 PL (StatSoft, Cracow, Poland). The mean value ± SD (for a normal distribution) was used. Student’s *t*-test and a chi-square analysis were performed to determine the differences between test scores by testing the date and performance on test items. A *p* < 0.05 was considered statistically significant, and all of the tests were two-tailed.

## 4. Results

### 4.1. Presentation of the Groups

The study group consisted of 20 girls. The patients were aged 5–18 years old at the moment of participating in the study. The mean age of the group was 10 years and 9 months old. Considering the study group, six of the girls had a positive family history of thyroid autoimmune diseases.

The control group consisted of 35 people. The patients were aged 2–18 years old at the moment of participating in the study. The mean age of the group was 10 years and 9 months old. In this group, five girls had a positive family history of thyroid autoimmune diseases.

### 4.2. Initial Symptoms of VLS

Information regarding symptoms and signs (present or preceding the diagnosis of VLS) was collected. The most common signs present in the girls were itching (60%, n = 12), soreness or a burning sensation (50%, n = 10), and excoriation (55%, n = 11).

When it comes to changes in the color of the skin and perineal mucosa, erythema (40%, n = 8) or/and pallor (40%, n = 8) were the most common. The typical figure “8” symptom occurred in 20% of the patients (n = 5). In 40% of girls, vulvar bleeding was reported (n = 7), among which three of the patients reported the presence of excoriation. Genital or urinary tract infections were reported in 25% of the girls (n = 5)

### 4.3. Diagnosis Time

Among the study group, four patients were diagnosed almost immediately after the onset of symptoms. However, in the remaining patients, this time was extended up to 36 months. The average time from the onset of symptoms to diagnosis was 10 months. The median age of onset of symptoms was 7 years old. The youngest patient was 2 years old, and the oldest 18 years old at the moment of diagnosis. Only two girls were diagnosed after menarche.

### 4.4. Family History

In the study group (n = 20), four girls had a documented personal history of asthma and/or allergies. Additionally, nine patients had a family history of autoimmune diseases among their first-degree relatives. The most commonly observed autoimmune diseases in this group were Hashimoto’s thyroiditis (in four girls) and rheumatoid arthritis (in three girls).

None of the patients in the control group had a personal history of allergies or asthma. However, a substantial part of the control group (in 22 girls) reported a family history of autoimmune diseases among their first-degree relatives. Among these, the most prevalent autoimmune conditions were Hashimoto’s thyroiditis (in nine patients), rheumatoid arthritis (in seven patients), and psoriasis (in six girls).

### 4.5. The Prevalence of Human IgG Antibodies in Study and Control Groups (Table 1)

#### 4.5.1. Antibodies against Thyroglobulin (TG)

In the study group (n = 20), only one of the girls had anti-TG antibodies, while in the control group (n = 35), six of them had anti-TG antibodies. Statistical significance was not achieved for the compared values (*p* = 0.37).

#### 4.5.2. Antibodies against Thyroid Peroxidase (Anti-TPO)

In the study group (n = 20), only one patient presented with anti-TPO antibodies, while in the control group (n = 35), three of them had anti-TPO antibodies. Comparing the obtained results, no statistical significance was found (*p* = 0.96).

**Table 1:** Frequency of the presence of characteristic antibodies for AITD in the studied population.

	Anti-TG (n)	Anti-TPO (n)
Study group (n = 20)	1	1
Control group (n = 35)	6	3
P (Chi-squared test)	(NS)	0.96 (NS)

### 4.6. The Prevalence of Human IgG Antibodies Compared to Onset of Symptoms

Only one patient in the study group had positive anti-TG. She was 9 years old at the moment of onset of symptoms, and was diagnosed at the age of 10 years and 6 months. In our study, the mean age of onset of symptoms with negative anti-TG was 6 years and 10 months (*p* = 0.593), and the mean age of diagnosis was 8 years (*p* = 0.563).

Anti-TPO antibodies were found in one patient. She was diagnosed at the age of 6 years at the moment of onset of symptoms. In the girls with negative anti-TPO, the mean age of onset of symptoms was 7 years (*p* = 0.798) and the mean age of diagnosis was 8 years and 3 months (*p* = 0.603).

## 5. Discussion

The coexistence of VLS and various autoimmune diseases, particularly thyroid autoimmune diseases, is a topic of increasing interest in the medical community [4,5,6,7,8]. This study aimed to explore the relationship between VLS and thyroid autoimmune diseases (AITD) in girls, with a particular focus on the presence of specific antibodies for AITD.

There is strong evidence indicating that autoimmunity plays a crucial role in the development of VLS. A greater proportion of VLS patients, in comparison to the general population, either suffer from an autoimmune-related condition, have a family history of autoimmune diseases, or present autoimmune antibodies. Among VLS patients, the most frequently observed autoimmune conditions include thyroiditis, alopecia areata, vitiligo, and pernicious anemia. Other less common associated conditions are autoimmune bowel disease, localized scleroderma/morphea, rheumatoid arthritis, systemic lupus erythematosus, and multiple sclerosis [11].

### 5.1. Symptoms

Patients with VLS commonly experience symptoms like itching, swelling, and a burning sensation in the vulva, along with pain, bleeding, and constipation. A significant proportion (86%) report itching, which often worsens at night, causing daytime fatigue in children. This itching can lead to skin tearing and bleeding. Some patients show no symptoms, and in 30% of girls with VLS, the diagnosis is delayed due to recurring infections [1,2,12,13].

Upon physical examination, distinct white skin lesions resembling a “Figure 8” or hourglass shape can be seen, especially around the labia and perianal region. The skin is atrophic and shiny. Other observations include erosions, scars, and bruises. Notably, the severity of symptoms does not always match the lesion’s size, meaning that even small lesions can cause significant discomfort [1,2,14].

In our study, the initial symptoms of VLS, such as itching, soreness, and excoriation, were consistent with the ones previously described in the literature. The presence of the figure “8” symptom in only 20% of the patients suggests that it may not be as common an indicator as previously thought, or it occurs in late-stage disease.

The course of vulvar lichen sclerosus varies widely, making it challenging to diagnose. Initial symptoms are often vague and can be overlooked by doctors who are not gynecologists or dermatologists. As a result, there can be a significant delay, sometimes spanning years, between the first examination and the confirmed diagnosis. According to Lagerstedt et al., only 16% of those with VLS receive a diagnosis in the disease’s early stages. On average, girls with vulvar lichen sclerosus start showing symptoms at 7.1 years of age, and there is typically a 1.3-year gap from when symptoms appear to when a diagnosis is made [15].

This information is consistent with our results. There is a delay between the onset of symptoms and the diagnosis. In our study, it was 10 months on average. Among the study group, four patients were diagnosed almost immediately after the onset of symptoms. However, in the remaining patients, this time was extended even up to 36 months.

The shorter average time of diagnosis might be a result of the relatively high availability of specialists in our region, especially access to pediatric gynecologists. However, as presented before, patients in which we found anti-TPO and anti-TG antibodies had their diagnosis delayed for 12 and 13 months, respectively.

The extended diagnosis time for a majority of the patients underscores the need for increased awareness and quicker diagnostic measures for VLS.

A correct diagnosis of vulvar lichen sclerosus in adolescence is crucial, because symptoms may reoccur in the postmenopausal age and can be associated with a higher risk of vulvar cancer [16,17].

### 5.2. Family History

The family history of thyroid autoimmune diseases was slightly higher in the study group (6/20) compared to the control group (5/35), suggesting a potential genetic predisposition in the study group.

The family history data are intriguing. While a significant proportion of the study group had a personal history of asthma and/or allergies, none in the control group reported such a history. This could suggest a potential link between VLS and other autoimmune or atopic conditions. However, the control group had a higher percentage of individuals with a family history of autoimmune diseases, particularly Hashimoto’s thyroiditis and rheumatoid arthritis. This raises questions about the broader genetic predispositions for autoimmune diseases and their potential interplay with VLS.

For instance, Aslanian et al. proved that there is a correlation between an intra-familial presence of certain haplotypes and anti-TPO, which emphasizes the link with thyroiditis [18]. In this study, the patients had their VLS diagnosed when they were already 30 years old and above.

The connection between LS (lichen sclerosus) and autoimmune disorders is more firmly established [19,20]. Individuals affected by LS show a higher prevalence of autoantibodies, particularly those targeting the thyroid, with a particular emphasis on anti-TPO [19,21,22].

In a study by Salim et al., interviews were carried out with 400 individuals who had LS, revealing that 15% of them had a family history of LS. It is plausible that a more comprehensive assessment could have uncovered additional instances of LS and autoimmune disorders within their relatives [23].

In contrast, Senturk et al. identified the same HLA alleles in four siblings (aged 7 to 16 years old) with LS, but these alleles were not found in their healthy sister. This observation suggests that genetic factors might be involved in the onset of LS. Interestingly, the authors noted that “none of the patients had autoimmune diseases,” even though three of them tested positive for anti-TPO, which could potentially indicate a heightened susceptibility to thyroid-related conditions in the future [24].

### 5.3. AITD and VLS

In terms of the specific antibodies for AITD, the results were somewhat unexpected. The presence of antibodies against thyroglobulin (TG) and thyroid peroxidase (anti-TPO) was relatively low in both groups. Only one girl in the study group had anti-TG antibodies, and similarly, only one had anti-TPO antibodies. In the control group, a slightly higher number had these antibodies, but the difference was not statistically significant. This suggests that while there might be a coexistence of VLS and thyroid autoimmune diseases, the presence of these specific antibodies might not be the best indicator of this relationship.

The delayed appearance of anti-TG and anti-TPO antibodies in relation to VLS might be caused by different pathophysiological timelines. While VLS symptoms might manifest earlier due to localized skin changes, the immune response against thyroid-specific antigens (like thyroglobulin and thyroid peroxidase) might take longer to develop and become detectable.

Research findings indicate that the occurrence of autoimmune thyroiditis (AT) tends to rise in prevalence among women as they grow older, a trend not observed in men. AT can impact a range of 5% to 20% of women of reproductive age and stands as the leading cause of hypothyroidism in the female population [25]. In contrast, AT is estimated to affect a considerably smaller proportion of children, with a prevalence ranging from 0.3% to 9.6% within the pediatric demographic. It is noteworthy that the condition is more frequently encountered in girls than in boys, with gender ratios ranging from 4:1 to 8:1, depending on the specific ethnic group [26].

Hu et al. observed that there was a significant difference in the prevalence of thyroid disease between the VLS group and non-VLS group (25.7% vs. 12.0%, *p* < 0.01) with a bivariate analysis. Additionally, thyroid disease was significantly associated with VLS when other factors were included in the multivariate regression model. The consistently observed relationship between VLS and thyroid disease may be at least partially attributable to the autoimmune dysregulation underlying both conditions [8].

The onset of VLS and the emergence of thyroid autoimmunity could potentially be influenced by environmental, genetic, and hormonal factors. It is possible that a singular factor may serve as a trigger of VLS symptoms, but the involvement of additional factors might be required to prompt the immune system into producing anti-TG and anti-TPO antibodies. The immune system operates in a multifaceted manner. The initial immune response to a trigger might lead to VLS symptoms, while a more specific or intensified response against thyroid antigens might develop later, leading to the production of anti-TG and anti-TPO antibodies. It is possible that thyroid autoimmunity starts at a subclinical level, where the immune system begins to target thyroid antigens but has not produced a sufficient quantity of antibodies to be detected in standard tests. As the autoimmune response intensifies over time, the antibody levels rise and become detectable.

Furthermore, VLS and thyroid autoimmune diseases could be interconnected within a spectrum of autoimmune disorders to which a patient might have a predisposition. VLS symptoms could potentially surface as the initial manifestation, with the development of thyroid autoimmunity occurring as part of the broader progression of autoimmune tendencies. Some of the triggering factors are especially similar, i.e., pregnancy and menopause. Those are periods in a woman’s life when hormonal fluctuations, including changes in estrogen and other hormones, may play an influential role [27,28].

It should be noted that our study is pediatric research, while most of the studies involve only adult patients [5]. For example, in a study involving 26 participants (25 females and one male) diagnosed with lichen sclerosus and 443 control subjects without autoimmune disorders, the authors conducted an assessment to detect the presence of antibodies related to thyroglobulin (Tg), thyroid cytoplasm, gastric parietal cells, and type I intrinsic factor. Notably, nearly half of the female patients tested positive for anti-thyroid cytoplasm antibodies (10 out of 25 (40%), with a significance of *p* < 0.001 compared to controls) or anti-gastric parietal cell antibodies (11 out of 25 (44%), with a significance of *p* < 0.001 compared to controls). The results of other tests were either negative or showed no statistical significance compared to the control group. Furthermore, it is worth mentioning that among those patients with anti-thyroid cytoplasm antibodies, eight were diagnosed with subclinical thyroiditis [21].

Given that VLS primarily affects girls and women, hormonal fluctuations and changes, especially those related to puberty, might influence the onset of VLS symptoms. The development of thyroid autoimmunity, on the other hand, might be influenced by a different set of hormonal changes or might require a longer period of hormonal influence.

## 6. Limitations

The number of patients participating in this study was relatively small, although it still represents the largest study conducted on a pediatric population to date. The COVID pandemic significantly hindered the recruitment of a larger number of patients, as some, due to health concerns, declined to participate in this study.

Another limitation of this study is the absence of thyroid hormone test results, such as TSH, FT3, and FT4. However, all patients are under the care of the gynecological clinic “Centrum Zdrowia Kobiety in Katowice”. Every girl, during their initial diagnosis of lichen sclerosus of the vulva, had thyroid hormone tests, and no disease was detected.

Additionally, each patient underwent routine neonatal screening for congenital thyroid deficiency, and all of them were found to be healthy. Up to this point, they had not been diagnosed with any thyroid disorders by their attending physicians.

Unfortunately, the mothers of the girls are not our patients, so VLS could have escaped our attention in their cases. Moreover, most of them are of reproductive age, and their hormonal status may suppress the symptoms.

## 7. Conclusions

In conclusion, while this study provides valuable insights into the potential relationship between VLS and thyroid autoimmune diseases in girls, the results also highlight the complexity of autoimmune conditions and their inter-relationships. The low prevalence of specific AITD antibodies in both groups suggests that other factors, possibly genetic or environmental, might play a more significant role in the coexistence of these conditions, which indicates the need for comprehensive patient monitoring. According to our best knowledge, this is the largest pediatric study on this topic conducted in Poland to date. Further research with larger sample sizes and a more diverse patient population might shed more light on these findings. Until further research is conducted, there is insufficient evidence to recommend routine screening for AITD in patients with VLS.

## Data Availability

The data presented in this study are available on request from the corresponding author. The data are not publicly available due to privacy or ethical reasons.

## References

[1] Jensen L.S., Bygum A. (2012). Childhood lichen sclerosus is a rare but important diagnosis. Dan. Med. J..

[2] Topal I.O., Sayilgan A.T., Kalcin S. (2017). An uncommon cause of vulval pruritus in childhood: *Lichen sclerosus*. Turk. Arch. Pediatr..

[3] Maronn M.L., Esterly N.B. (2005). Constipation as a Feature of Anogenital *Lichen sclerosus* in Children. Pediatrics.

[4] Bieber A.K., Steuer A.B., Melnick L.E., Wong P.W., Pomeranz M.K. (2020). Autoimmune and dermatologic conditions associated with lichen sclerosus. J. Am. Acad. Dermatol..

[5] Arduino P.G., Karimi D., Tirone F., Sciannameo V., Ricceri F., Cabras M., Gambino A., Conrotto D., Salzano S., Carbone M. (2017). Evidence of earlier thyroid dysfunction in newly diagnosed oral lichen planus patients: A hint for endocrinologists. Endocr. Connect..

[6] De Paola M., Batsikosta A., Feci L., Benedetti M., Bilenchi R. (2013). Granuloma Annulare, Autoimmune Thyroiditis, and Lichen Sclerosus in a Woman: Randomness or Significant Association?. Case Rep. Dermatol. Med..

[7] Guarneri F., Giuffrida R., Di Bari F., Cannavò S.P., Benvenga S. (2017). Thyroid Autoimmunity and Lichen. Front. Endocrinol..

[8] Hu J., Hesson A., Haefner H.K., Rominski S. (2020). The prevalence of self-reported medical comorbidities in patients with vulvar lichen sclerosus: A single-center retrospective study. Int. J. Gynecol. Obstet..

[9] Hu X., Chen Y., Shen Y., Tian R., Sheng Y., Que H. (2022). Global prevalence and epidemiological trends of Hashimoto’s thyroiditis in adults: A systematic review and meta-analysis. Front. Public Heal..

[10] Antonelli A., Ferrari S.M., Ragusa F., Elia G., Paparo S.R., Ruffilli I., Patrizio A., Giusti C., Gonnella D., Cristaudo A. (2020). Graves’ disease: Epidemiology, genetic and environmental risk factors and viruses. Best Pr. Res. Clin. Endocrinol. Metab..

[11] Corazza M., Schettini N., Zedde P., Borghi A. (2021). Vulvar Lichen Sclerosus from Pathophysiology to Therapeutic Approaches: Evidence and Prospects. Biomedicines.

[12] Powell J., Wojnarowska F. (2001). Childhood vulvar lichen sclerosus: An increasingly common problem. J. Am. Acad. Dermatol..

[13] Arlen A.M., Wang M., Vash-Margita A. (2020). Lichen Sclerosus in Prepubertal Girls: An Uncommon but Treatable Cause of Lower Urinary Tract Symptoms. Urology.

[14] Pranteda G., Muscianese M., Grimaldi M., Fidanza L., Narcisi A., Nisticò S., Bottoni U. (2013). *Lichen Sclerosus et Atrophicus* Induced by Carbamazepine: A Case Report. Int. J. Immunopathol. Pharmacol..

[15] Lagerstedt M., Karvinen K., Joki-Erkkilä M., Huotari-Orava R., Snellman E., Laasanen S. (2013). Childhood Lichen Sclerosus—A Challenge for Clinicians. Pediatr. Dermatol..

[16] Halonen P.M., Jakobsson M.I., Heikinheimo O., Riska A.E., Gissler M.V., Pukkala E.I. (2017). Lichen sclerosus and risk of cancer. Int. J. Cancer.

[17] Preti M., Borella F., Ferretti S., Caldarella A., Corazza M., Micheletti L., De Magnis A., Borghi A., Salvini C., Gallio N. (2023). Genital and extragenital oncological risk in women with vulvar lichen sclerosus: A multi-center Italian study. Maturitas.

[18] Aslanian F.M.N.P., Marques M.T.Q., Matos H.J., Pontes L.F.S., Porto L.C.S., Azevedo L.M.S., Filgueira A.L. (2006). HLA markers in familial Lichen Sclerosus. JDDG: J. der Dtsch. Dermatol. Ges..

[19] Marren P., Jell J., Charnock F., Bunce M., Welsh K., Wojnarowska F. (2006). The association between lichen sclerosus and antigens of the HLA system. Br. J. Dermatol..

[20] Harrington C.I., Dunsmore I. (1981). An investigation into the incidence of auto-immune disorders in patients with lichen sclerosus and atrophicus. Br. J. Dermatol..

[21] Goolamali S.K., Barnes E.W., Irvine W.J., Shuster S. (1974). Organ-specific Antibodies in Patients with Lichen Sclerosus. BMJ.

[22] Thomas R., Ridley C., Mcgibbon D., Black M. (1988). Lichen sclerosus et atrophicus and autoimmunity—a study of 350 women. Br. J. Dermatol..

[23] Salim A., Powell J., Gao X.-H., Wojnarowska F. (2002). Familial lichen sclerosus. Br. J. Dermatol..

[24] Şentürk N., Aydın F., Birinci A., Yildiz L., Cantürk T., Durupınar B., Turanlı A.Y. (2004). Coexistence of HLA-B*08 and HLA-B*18 in Four Siblings with Lichen sclerosus. Dermatology.

[25] Medici M., Porcu E., Pistis G., Teumer A., Brown S.J., Jensen R.A., Rawal R., Roef G.L., Plantinga T.S., Vermeulen S.H. (2014). Identification of Novel Genetic Loci Associated with Thyroid Peroxidase Antibodies and Clinical Thyroid Disease. PLoS Genet..

[26] Zois C., Stavrou I., Kalogera C., Svarna E., Dimoliatis I., Seferiadis K., Tsatsoulis A. (2003). High Prevalence of Autoimmune Thyroiditis in Schoolchildren After Elimination of Iodine Deficiency in Northwestern Greece. Thyroid.

[27] Tomer Y., Huber A. (2009). The etiology of autoimmune thyroid disease: A story of genes and environment. J. Autoimmun..

[28] Singh N., Ghatage P. (2020). Etiology, Clinical Features, and Diagnosis of Vulvar Lichen Sclerosus: A Scoping Review. Obstet. Gynecol. Int..

